# Patient experiences and outcomes from a novel multidisciplinary preoperative shared decision-making clinic

**DOI:** 10.1177/0310057X261427783

**Published:** 2026-04-20

**Authors:** Yiying (Sally) Tsang, Jia Hao Hui, Kate H Hurley, Sarah L Jones, Andrea Ditoro, Daire MacCormack, Andrew J Mackay

**Affiliations:** 1 Department of Anaesthesia and Perioperative Medicine, Northern Health, Epping, Australia; 2 Department of Geriatric Medicine, Northern Health, Epping, Australia; 3 Department of Medical Education, Melbourne Medical School, Melbourne, Australia; 4 Intensive Care Unit, Northern Health, Epping, Australia; 5 Division of Surgery, Northern Health, Epping, Australia

**Keywords:** Shared decision making, perioperative care, frailty, patient reported outcome measures

## Abstract

We established a novel multidisciplinary preoperative clinic in 2023 to support elderly, frail and/or comorbid patients to make decisions about surgical treatment based on their goals and values. We aimed to examine the characteristics, postoperative outcomes and quality of shared decision-making (SDM) for patients seen in this clinic in its first year. We collected data regarding patient demographics, preoperative status and postoperative outcomes via review of the medical record, and conducted a telephone interview to assess quality of SDM via the CollaboRATE-5 and Decision Regret Scales. Seventy-one patients attended the clinic in 2023, with a median age of 78 years. Eighty-six percent of patients had an American Society of Anesthesiologists Physical Status classification of 3 or 4, and 69% were frail (Clinical Frailty Scale of 4 or more). Sixty-five percent of patients elected to proceed with surgery and 35% elected not to proceed. At follow-up, approximately 14 months after initial SDM review, 58% of patients had had surgery, with a median postoperative hospital length of stay of four days, and a median of 25.5 days alive and at home within 30 days of surgery. Telephone interview was successfully conducted with 24 patients. The CollaboRATE-5 top score was reported by 63% of respondents, and the median Decision Regret Scale score was 7.5 (interquartile range 0–25) suggesting a high quality of SDM. In the first year of its operation, our multidisciplinary preoperative clinic delivered high-quality SDM to a cohort of high-risk surgical patients, with those proceeding to surgery generally experiencing favourable postoperative outcomes.

## Introduction

Because of demographic changes and advances in healthcare in recent decades, increasing numbers of patients with advanced age, frailty or multiple comorbidities are being considered for surgery.^1^ These patients often face difficult decisions, where surgery might offer benefits but is associated with higher perioperative risk and a more difficult recovery.^2,3^

Various models of specialist preoperative clinics have been established in recent years to target this high-risk population. These include the geriatrician-led Proactive Care of Older Patients Undergoing Surgery model comprising comprehensive geriatric assessment and optimisation^3,4^ and anaesthetist-led models such as the Complex Decision Pathway in Tauranga, New Zealand^5,6^ and the Shared Decision-Making Clinic at Peter MacCallum Cancer Centre in Melbourne.^7^ These clinics differ in personnel, format and patient selection, but often incorporate principles of shared decision-making (SDM), a collaborative approach that considers a patient’s values and goals alongside medical expertise regarding benefits and risks.^8,9^ SDM has been associated with decreased decisional conflict and increased decisional quality in planned surgery^10^ and decreased decisional regret.^11^

We set up a novel multidisciplinary preoperative clinic at our large metropolitan hospital in January 2023 to support elderly, frail and/or very comorbid patients in making decisions about surgical treatment based on their values and goals, following principles of SDM. We named our clinic the Complex Decision-Making (CDM) Clinic to clearly highlight the purpose of our clinic and the complexity of cases targeted, thus facilitating communication with multidisciplinary hospital teams. The clinic is staffed by an anaesthetist, a geriatrician and sometimes an intensivist, and supported by nursing and allied health staff. A notable feature of our clinic is its multidisciplinary nature, whereby different specialists review patients in a single clinic session, often in a joint consultation, and closely collaborate in management. Treating surgeons are also involved throughout the process from time of referral until after the final clinic appointment, although they are not usually present in the clinic.

As this was a novel clinic, we aimed to study the characteristics, postoperative outcomes and quality of SDM for patients who participated in this clinic in its first year of operation.

## Materials and methods

Following local ethics approval (Ref: 2024_non-HREC_32), data regarding patient demographics, preoperative status and postoperative outcomes, including length of stay, intensive care unit (ICU) admission and days alive and at home within 30 days of surgery (DAH_30_)^12^ were collected via review of the medical records for patients seen in the CDM Clinic between 1 January and 31 December 2023.

We assessed the quality of SDM using two measures: the CollaboRATE-5 scale, a patient-reported experience measure of SDM with psychometric validity,^13,14^ and the Decision Regret Scale^15^ (see Box 1). Median score and interquartile range (IQR) are presented for these scales. For the CollaboRATE-5 scale, the percentage of patients who reported the maximum possible score (‘top score’), is also presented.^14^ These scales were administered via telephone questionnaire between July and November 2024.

Box 1. **CollaboRATE-5 scale and Decision Regret Scale.**

Questions for the CollaboRATE-5 Scale:

1. How much effort was made to help you understand your health issues?

2. How much effort was made to listen to the things that matter most to you about your health issues?

3. How much effort was made to include what matters most to you in choosing what to do next?

Each question is scored from 0 (no effort) to 4 (maximal effort), with scores summed to give a total from 0 to 12. A higher score correlates to higher quality of patient-reported SDM.^13^

Questions for the Decision Regret Scale:

How do you feel about the following statements?

1. It was the right decision.

2. I regret the choice that was made.

3. I would go for the same choice if I had to do it over again.

4. The choice did me a lot of harm.

5. The decision was the right one.

Options: strongly agree, agree, neither agree nor disagree, disagree, strongly disagree, on a five-point Likert scale from 1 to 5, with questions 2 and 4 being reverse-scored, to give a sum between 5 and 25. This is then scaled to a range from 0 to 100 by subtracting 5, then multiplying by 5. A lower score corresponds to lower levels of decisional regret.

SDM: shared decision-making

As this is an exploratory study, descriptive analysis alone is used in presentation of study outcomes.

## Results

Seventy-one patients were reviewed in our CDM Clinic between 1 January and 31 December 2023. Their demographics and baseline characteristics are shown in Table 1. Patients were elderly, with a median age of 78 years. Eighty-six percent of patients had an American Society of Anesthesiologists Physical Status classification of 3 or 4, and 69% of patients were frail.

**Table 1.** Patient demographics (*N* = 71).

Age, years, median (IQR)	78 (70.5–84)
Sex, *n* (%)	
Male	44 (62%)
Female	27 (38%)
Cultural background, *n* (%)	
Born outside Australia	37 (52%)
Preferred language other than English	28 (39%)
ASA Physical Status classification	
2	10 (14%)
3	47 (66%)
4	14 (20%)
Clinical Frailty Score ⩾ 4, *n* (%)	49 (69%)

IQR: interquartile range; ASA: American Society of Anesthesiologists

**Figure 1.**
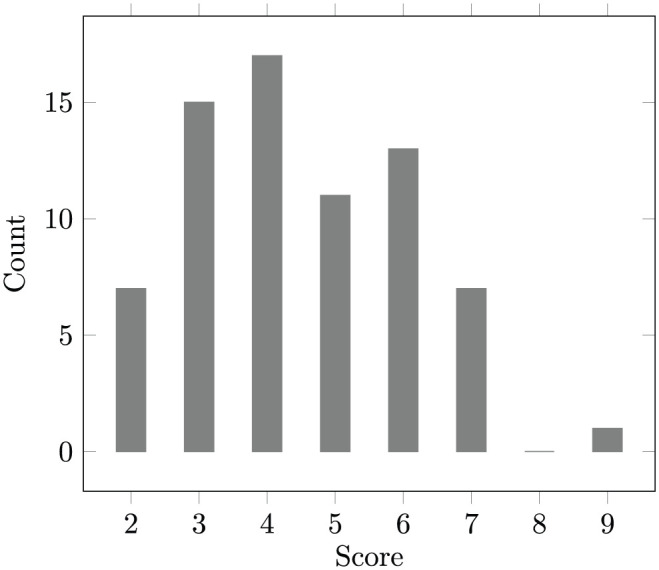
Rockwood Clinical Frailty Scale scores.

Figure 1 shows the distribution of their scores on the Rockwood Clinical Frailty Scale.^16^

Table 2 shows data related to CDM Clinic referrals and reviews. Following CDM Clinic review(s), 65% of patients decided to proceed with surgery, and 35% opted for non-surgical management. All surgical units at our hospital were represented amongst the patients except for otolaryngology.

**Table 2.** Characteristics of Complex Decision-Making Clinic assessment (*N* = 71).

Referral source, *n* (%)	
Surgical team	53 (75%)
Anaesthesia team	15 (21%)
Other medical team	3 (4%)
Patients seen in CDM Clinic by, *n* (%)	
Anaesthetist	65 (92%)
Geriatrician	55 (78%)
Intensivist	25 (35%)
Number of CDM Clinic reviews, *n* (%)	
One	51 (72%)
Two	20 (28%)
Outcome from Initial CDM Clinic assessment, *n* (%)	
Proceed to surgery	39 (55%)
Not for surgery	15 (21%)
Uncertain, for further CDM Clinic review	16 (23%)
Other	1 (1%)
Final decision from CDM Clinic, *n* (%)	
Proceed to surgery	46 (65%)
Non-operative management	25 (35%)

CDM: Complex Decision-Making

Follow-up of patients took place between 10 and 19 months after initial CDM Clinic visit. By then, 41 (58%) patients had proceeded to surgery. Six of the 46 patients from the CDM Clinic who decided to have surgery ultimately had non-operative management, and one patient from the CDM Clinic who opted for non-operative management had surgery. Table 3 shows a summary of postoperative outcomes. Median DAH_30_ was 25.5 days. The 30-day and one-year postoperative mortality rates were 4.9% and 12.2%, respectively. For patients who did not proceed to surgery, the one-year mortality rate, measured from initial CDM Clinic review, was 26.7%

**Table 3.** Postoperative outcomes in patients who proceeded to surgery (*N* = 41).

Mean acute hospital LoS, days, median (IQR)	4 (2–6)
ICU admission, *n* (%)	19 (46%)
Planned	18
Unplanned	1
ICU LoS, days, median (IQR)	1 (1–2)
DAH_30_, median (IQR)	25.5 (20–27)
Mortality, *n* (%)	
At 30 days	2 (4.9%)
At 12 months	5 (12.2%)

LoS: length of stay; IQR: interquartile range; ICU: intensive care unit; DAH_30_: days alive and at home within 30 days of surgery

**Figure 2.**
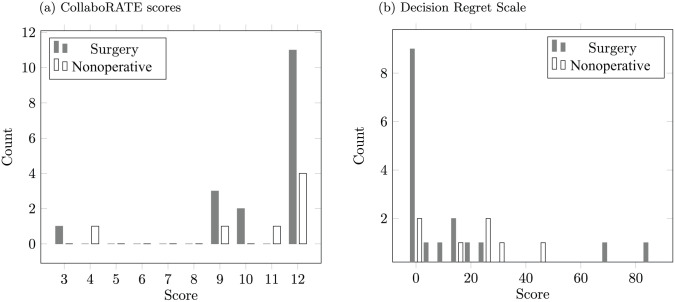
Follow-up assessment CollaboRATE scores (a) and Decision Regret Scale scores (b) for patients who did and did not have surgery.

Telephone follow-up was completed on average 14 months after initial clinic appointment. Telephone questionnaires were successfully conducted with 24 (34%) patients, 17 of whom had undergone surgery. Reasons for unsuccessful participation in telephone questionnaires are shown in Table 4. The CollaboRATE-5 mean score was 10.6, and 63% of respondents reported the CollaboRATE-5 top score of 12: 65% of patients who had surgery and 57% of patients who did not have surgery. The median Decision Regret Score was 7.5 (IQR 0–25) out of 100, corresponding to low levels of decisional regret. The Decision Regret Score was similar among patients who did and did not have surgery (Figure 2(a) and (b)).

**Table 4.** Reasons for not participating in follow-up phone call.

Patient does not consent	1
Unable to contact	8
Too unwell	7
Unable owing to language difficulties	14
Patient deceased	17
Total	47

## Discussion

In the first year of its operation, our CDM Clinic successfully identified a cohort of elderly, frail and comorbid patients being considered for surgery and supported them in making decisions about treatment according to their values and goals. Despite their high risk, 65% of patients elected to proceed with surgery, a rate comparable to other published data.^7^ Patients who proceeded to surgery generally experienced favourable postoperative outcomes, with short postoperative hospital and ICU lengths of stay, and a 30-day mortality in keeping with other high-risk surgical cohorts.^2^ We chose to include the DAH_30_ as a patient-centred measure of the quality of recovery after surgery.^12^ A recent large Australian retrospective cohort study of private health insurance data showed a median DAH_30_ of 27.1 days in all-comers, with lower DAH_30_ in older, more comorbid patients and patients treated in public hospitals.^17^ Thus, the median DAH_30_ of 25.5 days in our patient cohort indicates that, despite their elevated baseline risk, patients who proceeded with surgery after CDM review had a generally favourable postoperative trajectory.

At the same time, the CDM Clinic supported 35% of patients in pursuing a non-operative path, in accordance with their values and goals. These patients were able to avoid the burden of hospitalisation, surgery and recovery, where these were not thought to be concordant with their goals. More than a quarter of these patients had died within one year of initial CDM Clinic review, a rate much higher than the one-year postoperative mortality of patients who had had surgery. This is likely multifactorial and, in our view, illustrates two key points: first, many patients we see in high-risk preoperative clinics are in fact at the end of life,^18^ and more work is needed to better identify these patients; second, patients will often make decisions that lead to a reduced life expectancy but allow them to maximise quality of life in areas important to them, such as independence and time with loved ones. Although an economic analysis was outside the scope of this study, the choice of non-operative management by this cohort is also likely to have led to significant cost savings,^6^ especially considering the high rate of ICU utilisation in patients who did proceed to surgery.

Our telephone follow-up results suggest that our patient cohort, in general, experienced a high-level of SDM and low decisional regret. This is pleasing, given that previous research has shown that patients with frailty are more likely to experience greater decisional regret.^19^ We did not examine associations between types of surgery and decisional regret, as our numbers were small; this would be a useful question for future studies to address, since there is some signal that patients undergoing certain types of surgery, such as oncological surgery, report higher levels of regret.^11^

A key limitation of our study is the low proportion (34%) of patients who participated in telephone follow-up. By this time, almost a quarter of patients had died, and many others were too unwell or unable to be contacted. This might have led to selection bias, in which responses from patients with better outcomes were more likely to have been included in the results. Although it is common to follow up decisional regret at six to 12 months^20^ to allow patients time to reflect on the effects of their decisions, follow-up at an earlier time interval would likely have yielded a higher response rate in our cohort.

In addition, a large proportion of patients were unable to participate owing to language difficulties. This is consistent with the high level of cultural and linguistic diversity in our cohort but limits generalisability of our results. Moving forwards, we have adapted our ongoing data collection tool to include an electronic survey option, which can be done with assistance from friends or family, to help overcome this barrier.

We consider our clinic a ‘perioperative SDM clinic’ because it adheres to SDM principles in its communication approach and has the explicit aim of supporting patients in making decisions about surgical treatment. However, it ought to be acknowledged that there is currently no consensus definition of SDM in the perioperative space.^9^ Even in the broader literature, there is significant heterogeneity in measurement tools and outcomes studied.^21,22^ In a broad sense, SDM embodies the ideals of patient-centred care in a range of settings;^8^ in the Australian and New Zealand College of Anaesthetists Perioperative Framework, it is incorporated throughout the entire perioperative journey.^23^ However, perioperative SDM clinics targeting complex patients exist in a narrower space; further work is needed and is being done^9^ to define its components and streamline nomenclature.

Multidisciplinary collaboration is a cornerstone of our CDM service, and is a feature not universally shared by other SDM clinics. Evidence-based decisions can be difficult for complex and frail patients because they often exhibit individual factors that are not well represented in research.^24^ Their comorbidities often require expertise from multiple specialties, and their frailty and complexity mean that small decisions can have large impacts on their life trajectory. A multidisciplinary approach that views issues from multiple angles has been posited to be ‘the best available decision-making tool’ for very complex or frail patients.^24^ Following the well-established evidence for comprehensive geriatric assessment in orthogeriatric care, there is growing evidence to support perioperative geriatrician involvement in non-orthogeriatric surgical settings such as in the CDM Clinic.^25^ Since 2024, our CDM Clinic has also conducted monthly multidisciplinary meetings to facilitate closer collaboration with surgeons and other specialists, in line with proposed best practice.^9^

In addition to decision-making and physician involvement in medical optimisation, multidisciplinary collaboration has also benefited our CDM Clinic patients in other ways. For patients proceeding to surgery, ICU involvement in the CDM Clinic has enabled enhanced perioperative planning, both in informing patients about and facilitating access to high-dependency care, as well as promoting continuity of care in the immediate perioperative period. The high proportion of planned ICU admissions reflects the important support of ICU colleagues to our service and their recognition that the early postoperative phase is crucial in optimising postoperative outcomes. Likewise, geriatrician involvement has often translated to continuity of care in the post-acute and/or community phase, where interventions can be facilitated that optimise patients’ recovery and function after they leave hospital.

Finally, multidisciplinary collaboration in our clinic has led to benefits for healthcare teams. Information-sharing between specialties has led to increased perioperative expertise among clinicians—for geriatricians, a more nuanced understanding of anaesthetic risk, and for anaesthetists, an increased appreciation of how the perioperative period fits into patients’ life trajectories. Collaboration in and around our CDM Clinic has improved professional relationships between perioperative specialties and reduced the burden of difficult decision-making shouldered by individuals. Collaborative decision-making might reduce regret among surgeons after adverse events^26^ and thereby contribute to the wellbeing of healthcare teams. There is scant literature regarding impacts of multidisciplinary shared decision-making on promoting wellbeing and reducing moral injury in healthcare providers; further study in this area would be beneficial.

## Conclusion

In the first year of its operation, our novel, multidisciplinary, preoperative SDM clinic successfully assisted a cohort of elderly, frail and comorbid patients to make decisions about surgical treatment based on their values and goals. Patients who proceeded to surgery generally experienced favourable postoperative outcomes. Patients who participated in telephone follow-up reported high quality of SDM and low decisional regret. Despite its limitations, our study adds to the limited and much needed body of evidence on perioperative SDM and patient-reported outcome measures.
